# Renal and Skeletal Anomalies in a Cohort of Individuals With Clinically Presumed Hereditary Nephropathy Analyzed by Molecular Genetic Testing

**DOI:** 10.3389/fgene.2021.642849

**Published:** 2021-05-26

**Authors:** Michaela Stippel, Korbinian M. Riedhammer, Bärbel Lange-Sperandio, Michaela Geßner, Matthias C. Braunisch, Roman Günthner, Martin Bald, Miriam Schmidts, Peter Strotmann, Velibor Tasic, Christoph Schmaderer, Lutz Renders, Uwe Heemann, Julia Hoefele

**Affiliations:** ^1^Institute of Human Genetics, Klinikum rechts der Isar, School of Medicine, Technical University of Munich, Munich, Germany; ^2^Department of Nephrology, Klinikum rechts der Isar, School of Medicine, Technical University of Munich, Munich, Germany; ^3^Division of Pediatric Nephrology, Dr. v. Hauner Children's Hospital, Ludwig-Maximilians University, Munich, Germany; ^4^Pediatric Nephrology, University Children's Hospital, Tübingen, Germany; ^5^Pediatric Nephrology, Olgahospital, Klinikum Stuttgart, Stuttgart, Germany; ^6^Center for Pediatrics and Adolescent Medicine, University Hospital Freiburg, Freiburg University Faculty of Medicine, Freiburg, Germany; ^7^Pediatric Nephrology, Children's Hospital, München-Klinik Schwabing, Klinikum rechts der Isar, Technical University of Munich, Munich, Germany; ^8^Medical Faculty of Skopje, University Children's Hospital, Skopje, Macedonia

**Keywords:** hereditary nephropathy, CAKUT, podocytopathy, FSGS, SRNS, ciliopathy, skeletal anomaly

## Abstract

**Background:** Chronic kidney disease (CKD) in childhood and adolescence occurs with a median incidence of 9 per million of the age-related population. Over 70% of CKD cases under the age of 25 years can be attributed to a hereditary kidney disease. Among these are hereditary podocytopathies, ciliopathies and (monogenic) congenital anomalies of the kidney and urinary tract (CAKUT). These disease entities can present with a vast variety of extrarenal manifestations. So far, skeletal anomalies (SA) have been infrequently described as extrarenal manifestation in these entities. The aim of this study was to retrospectively investigate a cohort of individuals with hereditary podocytopathies, ciliopathies or CAKUT, in which molecular genetic testing had been performed, for the extrarenal manifestation of SA.

**Material and Methods:** A cohort of 65 unrelated individuals with a clinically presumed hereditary podocytopathy (focal segmental glomerulosclerosis, steroid resistant nephrotic syndrome), ciliopathy (nephronophthisis, Bardet-Biedl syndrome, autosomal recessive/dominant polycystic kidney disease), or CAKUT was screened for SA. Data was acquired using a standardized questionnaire and medical reports. 57/65 (88%) of the index cases were analyzed using exome sequencing (ES).

**Results:** 8/65 (12%) index individuals presented with a hereditary podocytopathy, ciliopathy, or CAKUT and an additional skeletal phenotype. In 5/8 families (63%), pathogenic variants in known disease-associated genes (1x *BBS1*, 1x *MAFB*, 2x *PBX1*, 1x *SIX2*) could be identified.

**Conclusions:** This study highlights the genetic heterogeneity and clinical variability of hereditary nephropathies in respect of skeletal anomalies as extrarenal manifestation.

## Introduction

Chronic kidney disease (CKD) in childhood and adolescence occurs with a median incidence of 9 per million of the age-related population (Harambat et al., 2012). A hereditary (monogenic) nephropathy accounts for over 70% of CKD cases with onset under 25 years of age (Vivante and Hildebrandt, 2016). Hereditary nephropathies comprise a group of clinically and genetically heterogeneous kidney disorders representing a significant risk for the development of ESRD (end-stage renal disease) (Stavljenic-Rukavina, 2009). About 6% of children up to the age of 14 years on renal replacement therapy (RRT) have a diagnosed hereditary nephropathy. Additionally, congenital anomalies of the kidney and urinary tract (CAKUT) and cystic kidney diseases, in which various monogenic disorders have been identified, are present in more than 50% of children up to the age of 14 years on RRT (Chesnaye et al., 2014). Hence, hereditary nephropathies, which can occur in isolated or syndromic forms, pose a substantial burden on mortality and morbidity.

More than 200 clinically distinct syndromes have been described to involve renal/urinary tract malformations (van der Ven et al., 2018). However, skeletal anomalies (SA) as extrarenal feature of hereditary nephropathies are infrequently described in the literature, but can be part of syndromic forms of hereditary podocytopathies (e.g., focal segmental glomerulosclerosis, steroid resistant nephrotic syndrome), ciliopathies (e.g., nephronophthisis), or CAKUT (Solomon, 2011; Romani et al., 2013; Priya et al., 2016; Riedhammer et al., 2017; Titieni and König, 2018; Haffner and Petersen, 2019; König et al., 2019). The frequency of the phenotypic combination of renal and skeletal anomalies depends on the underlying disease/disease entity and can be observed, for example, in individuals with CAKUT in up to 50% (Stoll et al., 2014). This is not surprising, as both the skeleton and kidney originate from the mesoderm and their development share important pathways, such as Wnt- and hedgehog signaling (Barker et al., 2014; Krause et al., 2015).

This study highlights eight individuals with extrarenal features of SA in a cohort of individuals with clinical suspicion of a hereditary podocytopathy, ciliopathy, or CAKUT in which molecular genetic testing had been performed.

## Materials and Methods

The study was approved by the local Ethics Committee of the Technical University of Munich and performed according to the standard of the Helsinki Declaration of 2013. Written informed consent was obtained by all participants or their legal guardians. All individuals were clinically examined by pediatric or adult nephrologists.

### Study Cohort

All individuals were referred to the Institute of Human Genetics of the Klinikum rechts der Isar of the Technical University of Munich, a tertiary care center. Clinical data was acquired using a standardized questionnaire and medical reports. The study cohort encompassed 65 unrelated index individuals (index cases; 42/65 [65%] of Non-Finnish European descent) with a clinically presumed hereditary podocytopathy, ciliopathy, or CAKUT and was screened for skeletal anomalies. The included individuals are part of a larger cohort of ~300 families with clinically presumed hereditary kidney disease (“NephroGen”) collected between 2015 and 2018. The NephroGen cohort additionally contains families with clinically presumed hereditary kidney diseases like type-IV-collagen-related nephropathy (Alport syndrome, thin basement membrane nephropathy), tubulopathies, and autosomal dominant tubulointerstitial kidney disease (ADTKD), which were not included in this study.

Molecular-genetic analysis was performed primarily by exome sequencing (ES). However, in selected cases—depending on the clinical phenotype—single-gene Sanger sequencing, single nucleotide polymorphism (SNP) array or multiplex ligation-dependent probe amplification (MLPA) was performed (see Results).

### Exome Sequencing

DNA was extracted from peripheral blood using either the Gentra Puregene Blood Kit (Qiagen, Hilden, Germany) or the Chemagic 360 (Perkin Elmer, Waltham) according to the manufacturer's instructions. ES was performed using a Sure Select Human All Exon 60 Mb V6 Kit (Agilent) and a HiSeq4000 (Illumina) or a Sure Select Human All Exon 50 Mb V5 Kit (Agilent) and a HiSeq2500 (Illumina) (Kremer et al., 2017). Mitochondrial DNA was derived from off-target exome reads (Griffin et al., 2014). Reads were aligned to the human reference genome (UCSC Genome Browser build hg19) using Burrows-Wheeler Aligner (v.0.7.5a). Detection of single-nucleotide variants and small insertions and deletions (indels) was performed with SAMtools (version 0.1.19). ExomeDepth was used for the detection of copy number variants (CNVs). A noise threshold of 2.5 was accepted for diagnostic analysis (Plagnol et al., 2012). Called CNVs were visualized by the Integrative Genomics Viewer (IGV, https://software.broadinstitute.org/software/igv/) to check for sufficient coverage of the inspected region and plausibility of the CNV. CNVs were compared with publicly available control databases like the Genome Aggregation Database (gnomAD, https://gnomad.broadinstitute.org/about), the Database of Genomic Variants (DGV, http://dgv.tcag.ca/dgv/app/home) and databases for pathogenic CNVs like DECIPHER (https://decipher.sanger.ac.uk/) and ClinVar (https://www.ncbi.nlm.nih.gov/clinvar/). Rating was done according to American College of Medical Genetics (ACMG) guidelines and the recommendation of the ACGS (Richards et al., 2015; Ellard et al., 2019; Riggs et al., 2020). For the analysis of *de novo*, autosomal dominant and mitochondrial variants, only variants with a minor allele frequency (MAF) of <0.1% in an in-house database (“Munich Exome Server”) containing over 21,000 exomes were considered. For the analysis of autosomal recessive and X-linked variants (homozygous, hemizygous or putative compound heterozygous) variants with a MAF of <1.0% were considered. As there are pathogenic alleles in hereditary nephropathies (HNs) with a MAF of more than 1.0% like the *NPHS2* p.Arg229Gln allele (also known as p.R229Q), in unsolved cases, an additional search for recessive and X-linked variants a MAF up to 3% was accepted. The p.Arg229Gln allele is known to cause steroid resistant nephrotic syndrome when *in trans* with specific 3′ *NPHS2* variants (Tory et al., 2014; Miko et al., 2018). Identified variants were compared with publicly available databases for pathogenic variants like ClinVar, the Human Gene Mutation Database (HGMD^®^, http://www.hgmd.cf.ac.uk) and the Leiden Open Variation Database (LOVD, https://www.lovd.nl). Only variants rated as “likely pathogenic” or “pathogenic” according to the classification of the ACMG and with a genotype in agreement with the mode of inheritance led to the designation “solved case” (Kearney et al., 2011; Richards et al., 2015; Riggs et al., 2020).

### Sanger Sequencing, SNP Array, and MLPA

Molecular genetic testing of selected cases or segregation analysis of identified variants in parents and affected relatives was performed by Sanger sequencing, SNP array or MLPA as appropriate. Oligonucleotide primer sequences used for Sanger sequencing are available upon request. DNA alignment and sequence variant analysis were carried out using the Sequence PilotCE software (JSI Medical Systems GmbH, Kippenheim, Germany) and compared to EMBL (European Molecular Biology Laboratory) and GenBank databases as well as our in-house database. For SNP array, Affymetrix^®^ CytoScanTM 750 K Array (Affymetrix^®^ Inc., Santa Clara, CA, USA) with an average space between two oligonucleotides of 4 kilobases (kb) was used. Scanning was performed by the Affymetrix^®^ GeneChip Scanner 3000 7G (resolution 0.51–2.5 μm). The data analysis was conducted using the Affymetrix^®^ Chromosome Analysis Suite Software (ChAS), version 3.0, hg19. For MLPA, the SALSA MLPA kit P241-D2 Kit (maturity-onset diabetes of the young, renal cysts and diabetes syndrome, *HNF1B*) was purchased from MRC-Holland (Amsterdam, the Netherlands) and performed according to the manufacturer's instructions. Fragment analysis was conducted using GeneMarker software (www.softgenetics.com, Soft Genetics, State College, PA, USA).

Molecular genetic results were communicated to the families either via the referring clinician or as part of genetic counseling.

## Results

**Figure 1:**
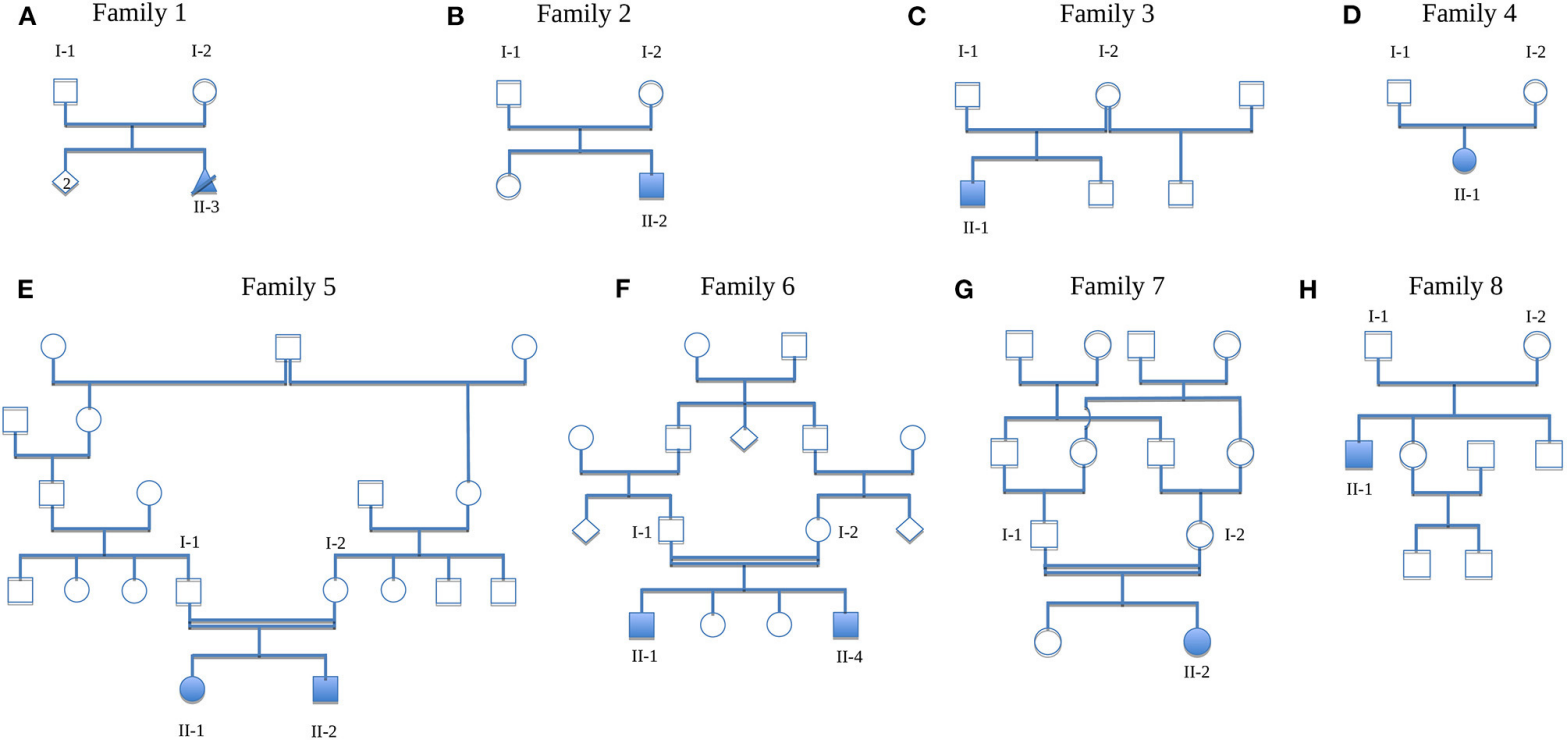
Pedigrees of the families. **(A)** Family 1 (HN-F18), **(B)** Family 2 (HN-F151), **(C)** Family 3 (HN-F75), **(D)** Family 4 (HN-F80), **(E)** Family 5 (HN-F191), **(F)** Family 6 (HN-F198), **(G)** Family 7 (HN-F241), **(H)** Family 8 (HN-F305). Solid symbols, affected individuals; circles, females; squares, males; crossed triangle, miscarriage; diamond, unknown gender; double lines, consanguinity.

The total cohort of 65 index individuals comprised 18 (28%) individuals with the clinical picture of a hereditary podocytopathy (one exception of an index case [HN-F203-II-2] who was initially classified as having a type-IV-collagen-related nephropathy based on the clinical phenotype and later reclassified as hereditary podocytopathy on genetic diagnosis; see Supplementary Table 1), 20 (31%) with a ciliopathy, and 27 (41%) with CAKUT. A total of eight individuals (12%) displayed one or more skeletal anomalies additionally to their renal phenotype (Figure 1, Table 1, family description below).

**Table 1:** Overview of the index cases and also affected siblings including a detailed description of phenotype and genotype.

**Individuals** **and sex(index, further affected individuals)**	**Genetically solved**	**Gene(transcript)**	**Inheri-tance**	**Variant with cDNA and protein position and inheritance (in parenthesis)**	**Chromosomal position (hg19)**	**Zygosity**	**ACMG rating**	**Applied ACMG criteria for variant interpretation/** **ACMG CNV score**^**#**^	**ClinVar**^**##**^	**Clinical tentative diagnosis or genetic diagnosis with MIM number in parenthesis (if case was solved genetically)**	**Age at first presentation**	**Renal** **phenotype**	**Skeletal phenotype**	**Additional phenotype**
Family 1(HN-F18)II-3male	No	-	-	-	-	-	-	-	-	CAKUT	24th week of gestation	Bilateral renal agenesis	Micrognathia	Pes equinovarus, hypoplastic nasal wings, flat philtrum, short and pointed fingers, knuckle pads
Family 2 (HN-F51) II-2male	Yes	*MAFB*(NM_005461.4)	AD	c.184A>G,p.(Thr62Ala)(*de novo*)	chr20:g.3931 7307T>C	Het	Pathogenic	PS2 PM2 PM5 PP3 PP4	n.l.	Multicentric carpotarsal osteolysis syndrome (166300)	2 years	Small right kidney, proteinuria	Shortened ulna and fingers	Broad root and spherical tip of the nose, discreet epicanthus, long philtrum, narrow upper lip, thick and blue thumb base, the doughy hands, hypoplastic toes
Family 3 (HN-F75) II-1male	Yes	*PBX1*(NM_002585.3)	AD	c.413_419delp.(Gly138Valfs^*^40)(*de novo*)	chr1:g.164761 878_16476 1884del	Het	Pathogenic	PVS1 PS2 PM2	n.l.	CAKUTHED(617641)	At birth	Bilateral renal dysplasia	Hypoplastic claviculae, cleidocranial dysplasia, clinodactyly and brachydactyly of the little finger	Intellectual disability
Family 4 (HN-F80) II-1female	No	*SIX2*(NM_016932.5)	AD	Deletion -at least 2.9 kb involving the complete exonic regions of SIX2^*^(*de novo*)	Approx. chr2:g.45233309-45236248	Het	Pathogenic^###^	1,00	n.l.	Unknown syndromic kidney disorder	6 months	Bilateral renal hypodysplasia	Frontonasal dysplasia with retracted nasal root, dispropor tionate short stature	Decreased vision in one eye
Family 5 (HN-F191) II-1female	No	-	-	-	-	-	-	-	-	Bardet-Biedl-like phenotype	At birth	Chronic kidney disease, tubule-interstitial scarring, renal tubular ectasia	Disproportionate short stature, bilateral genu vara	Mitral valve insufficiency, atrial septal defect, persistent ductus arteriosus, pulmonary hypertension, bronchial hyperreactivity
Family 5(HN-F191) II-2male	No	-	-	-	-	-	-	-	-	Bardet-Biedl-like phenotype	At birth	Chronic kidney disease	Short stature	Recurrent obstructive bronchitis, hepatomegaly
Family 6 (HN-F198) II-1male	Yes	*BBS1*(NM_024649.4)	AR	Complex allele c.[831-3C>G; 1285C>T];[831-3C>G;1285C>T], p.[?;Arg429^*^]; [?;Arg429^*^](maternal, paternal)	chr11:g.66290 924C>G and chr11:g.66294 224C>T	Hom	Pathogenic	PS4_supporting PM2 PM3 PP3andPVS1 PS4_supporting PM2	c.831-3C> G: patho genic and c.1285C>T: likely pathogenic	Bardet-Biedl syndrome 1(209900)	1 year	Right vesicoureteral reflux	Polydactyly of the feet, oligodactyly right hand, left split hand	Microcephaly, developmental delay, anal atresia, severely impaired vision and oculocutaneous albinism
Family 6(HN-F198) II-4male	Yes	*BBS1*(NM_024649.4)	AR	Complex allele c.[831-3C>G; 1285C>T];[831-3C>G;1285C>T], p.[?;Arg429^*^]; [?;Arg429^*^](maternal, paternal)	chr11:g.66290 924C>G and chr11:g.66294 224C>T	Hom	Pathogenic	PS4_supporting PM2 PM3 PP3andPVS1 PS4_supporting PM2	c.831-3C> G: patho genic and c.1285C>T: likely pathogenic	Bardet-Biedl syndrome 1(209900)	At birth	Hypoplastic horseshoe kidney	Hexadactyly of both hands and feet, narrow thorax	Liver fibrosis, anal atresia
Family 7(HN-F241) II-2female	No	-	-	-		-	**-**	**-**	**-**	Dysostosis multiplex	2 years	Right sided double kidney	Short stature	Coarse facial features
Family 8(HN-F305) II-1male	yes	*PBX1*(NM_002585.3)	AD	c.661G>T, p.(Glu221^*^)(*de novo*)	chr1:g.164769086G>T	Het	Pathogenic	PVS1 PS2 PM2	n.l.	CAKUTHED(617641)	1 year	Right renal agenesis, left renal hypoplasia	Short stature	Choanal atresia, facial dysmorphic signs

# *See (Richards et al., 2015; Ellard et al., 2019; Riggs et al., 2020). In general, PVS1 indicates a very strong level of pathogenicity, PS designations indicate strong evidence of pathogenicity, PM designations indicate a moderate level of pathogenicity, and PP designations indicate supporting evidence of pathogenicity*.

## *Based on https://www.ncbi.nlm.nih.gov/clinvar/ (ClinVar ratings solely by our institute are not listed)*.

### *This is a pathogenic variant. However, haploinsufficiency of SIX2 has been associated with frontonasal dysplasia with ptosis and hearing loss but is not known to be associated with renal hypodysplasia so far, hence no exact genetic diagnosis (see Discussion)*.

*AD, autosomal dominant; AR, autosomal recessive; CAKUT, congenital anomalies of the kidney and urinary tract; CAKUTHED, congenital anomalies of kidney and urinary tract syndrome with or without hearing loss, abnormal ears, or developmental delay; het, heterozygous; hom, homozygous; n.l., not listed*.

**Figure 2:**
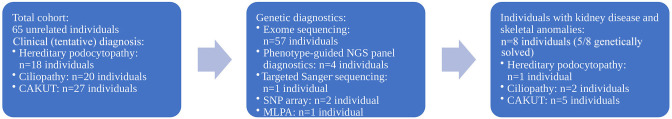
Flowchart of the cohort. CAKUT, congenital anomalies of the kidney and urinary tract; MLPA, multiplex ligation-dependent probe amplification; NGS, next-generation sequencing; SNP, single nucleotide polymorphism.

In 57/65 (88%) index cases, ES was performed. 8/65 (12%) index cases were analyzed either by phenotype-guided panel diagnostics (4/8; 50%), single-gene Sanger sequencing (1/8; 13%), SNP array (2/8; 25%), or MLPA (1/8; 13%) (Figure 2). 22/65 (34%) index cases could be genetically solved (i.e., [likely] pathogenic variant with a fitting genotype identified).

The eight index cases with an additional skeletal phenotype are presented as follows. The remaining 57 index cases are not specifically featured in this paper. Phenotypic and genotypic data of these cases can be found in the Supplementary Table 1.

### Family 1 (HN-F18; CAKUT)

In Family 1, the third, unborn male child (II-3; Non-Finnish European descent) was pre-natally diagnosed with bilateral renal agenesis. The pregnancy was therefore pre-maturely terminated. The fetus presented with dysmorphological features such as knuckle pads at the proximal interphalangeal joint of both hands, short and pointed fingers and pes equinovarus. Other conspicuous facial features were hypoplastic nasal wings, a flat philtrum and micrognathia. There were no known affected relatives, parents and siblings of the index individual were healthy.

A previously conducted chromosome analysis revealed no pathological findings. Afterwards, trio ES was performed on the index and the parents, which also was inconspicuous.

### Family 2 (HN-F51; Hereditary Podocytopathy)

The son (F2-II-2; Non-Finnish European descent) of healthy parents presented with proteinuria at the age of 2 years. He additionally had shortened ulna and fingers, which were broad at the basis and tapered to the end. The base of the thumb was thickened and blue, the hands were doughy and toenails and toes hypoplastic. Further signs of dysmorphia were a broad root and a spherical tip of the nose, discreet epicanthus, a long philtrum, and a narrow upper lip. There were no affected relatives.

Proband-only ES identified a heterozygous pathogenic missense variant NM_005461.4:c.184A>G, p.(Thr62Ala) in *MAFB*. Targeted Sanger sequencing of the parents confirmed its *de novo* status. *MAFB* has been associated with multicentric carpotarsal osteolysis syndrome (MTCO, MIM 166300). It encodes a transcription factor which impacts the differentiation and activation of osteoclasts as well as renal development. The disease occurs very rarely and it features aggressive osteolysis especially in the area of carpal and tarsal bones. It is frequently associated with a progressive kidney failure (Zankl et al., 2012).

### Family 3 (HN-F75; CAKUT)

This case has already been published (Riedhammer et al., 2017). The renal phenotype of the male index case in this family (II-1; Non-Finnish European descent) consisted of bilateral renal dysplasia which was first seen at the age of 3 months. At the age of 4 years, he had already developed renal insufficiency. Furthermore, different skeletal anomalies like hypoplastic claviculae, cleidocranial dysplasia, clinodactyly and brachydactyly of the little finger as well as a narrow thorax and shoulders were present. Moreover, he had intellectual disability. There were no other affected family members.

First, SNP array analysis was performed but did not reveal any causative microdeletions or microduplications. Afterwards, proband-only ES was performed in the index case identifying the pathogenic frameshift variant NM_002585.3:c.413_419del, p.(Gly138Valfs^*^40) in *PBX1*. *PBX1* is a known CAKUT disease-associated gene (MIM 617641) (Heidet et al., 2017). The parents were examined using Sanger sequencing, which confirmed the *de novo* status of this variant.

### Family 4 (HN-F80; CAKUT)

This female individual (II-1; Non-Finnish European descent) of Family 4 had developed ESRD already in childhood as a result of renal hypodysplasia. Extrarenal manifestations were decreased vision in one eye, frontonasal dysplasia with retracted nasal root, and short stature due to skeletal dysplasia. Her parents were healthy as well as other family members.

Trio ES was conducted and identified a *de novo* heterozygous deletion of at least 2.9 kb including the complete exonic regions of *SIX2* (approx. chr2:g.45233309-45236248). While *SIX2* missense variants have been associated with renal hypodysplasia, *SIX2* haploinsufficiency has not been linked to a renal phenotype so far but to frontonasal dysplasia, ptosis, and hearing loss (Weber et al., 2008; Guan et al., 2016; Henn et al., 2018).

### Family 5 (HN-F191; Ciliopathy)

Family 5 featured two siblings with a syndromal phenotype, fitting best into the spectrum of ciliopathies. Their parents were healthy, of Turkish origin and consanguineous. The female index case (II-1) as well as her younger brother (II-2) showed various congenital anomalies. The girl presented with cardiovascular anomalies such as mitral valve insufficiency, atrial septal defect, persistent ductus arteriosus and pulmonary hypertension. Furthermore, she suffered from bronchial hyperreactivity, which lead to recurrent pneumonia. Noticeable skeletal anomalies were disproportionate short stature and bilateral genu vara. The renal phenotype was designated as nephropathy of unclear origin. A biopsy revealed glomerular and tubulo-interstitial scarring as well as minimal tubular ectasia of the cortex. Her clinical phenotype was comparable with a Bardet-Biedl-like syndrome. Her brother showed similar anomalies. He presented with pulmonary symptoms such as recurrent, partly obstructive bronchitis and short stature. Furthermore, hepatomegaly was observed in him.

Using ES in the female index individual, no causative variants in known disease-associated genes could be identified.

### Family 6 (HN-F198; Ciliopathy)

In Family 6, two individuals had the clinical tentative diagnosis of Bardet-Biedl syndrome. The male index individual (II-1) as well as his younger brother (II-4) were affected. Their parents were healthy, of Pakistani origin and consanguineous. Other relatives did not show any signs of kidney disorder. The index individual had already developed ESRD at the age of 4 years as a consequence of recurrent pyelonephritis with abscess formation due to vesicoureteral reflux on the right side. Kidney transplantation was performed at the age of 4 years. In addition to the renal phenotype, the individual had oculocutaneous albinism with serious vision problems (no signs for retinal dystrophy), severe intellectual disability, epilepsy, multiple intra-abdominal abscesses and anal atresia. He also had Factor V Leiden thrombophilia. Concerning the skeletal appearance, he had bilateral fibular polydactyly, oligodactyly of the right hand and a cleft hand on the left side. His younger brother had a hypoplastic-cystic horseshoe kidney. He additionally presented with liver cirrhosis and anal atresia. Similar to the index individual, he had skeletal anomalies such as hexadactyly of both hands and feet. Furthermore, a slim thorax and lateralized mammilla were observed.

Molecular genetic diagnostics using duo ES was performed. Both affected individuals had a pathogenic homozygous complex allele NM_024649.4:c.[831-3C>G;1285C>T];[831-3C>G;1285C>T], p.[?;Arg429^*^];[?;Arg429^*^] in *BBS1*. The parents were heterozygous carriers for the complex allele. *BBS1* is associated with autosomal recessive Bardet-Biedl syndrome (MIM 209900) (Mykytyn et al., 2002).

### Family 7 (HN-F241; CAKUT)

In Family 7, the healthy parents were of Egyptian origin and consanguineous. One of the daughters (II-2) presented with double kidney on the right side as well as dysostosis multiplex. Coarse facial features and short stature were striking in appearance. Both parents and the sister of the affected individual as well as other relatives were healthy.

Proband-only ES was performed and was inconspicuous.

### Family 8 (HN-F305; CAKUT)

The son (II-1; Non-Finnish European descent) of healthy parents of Family 8 showed dysplasia of the right and hypoplasia of the left kidney. Extrarenal manifestations were choanal atresia, short stature, and facial dysmorphic signs. At the age of 6 years he underwent the first kidney transplantation, a second kidney was transplanted at the age of 11 years. Consanguinity or further affected relatives were not reported.

Trio ES was conducted and revealed the pathogenic heterozygous *de novo* variant NM_002585.3:c.661G>T, p.(Glu221^*^) in *PBX1*. Further information on *PBX1* are already mentioned in Family 3.

## Summary

Of the total 65 index cases, SA were present in 1/18 (6%) individuals with a hereditary podocytopathy, 2/20 (10%) individuals with a ciliopathy, and 5/27 (19%) individuals with CAKUT.

In terms of diagnostic yield in individuals with an additional extrarenal phenotype of SA, 5/8 (63%) cases could be genetically solved: 1/5 (20%) with a hereditary podocytopathy, 1/5 (20%) with a ciliopathy, and 3/5 (60%) with CAKUT (Figure 2). Of note, concerning Family 4, haploinsufficiency of *SIX2* has not been associated with a renal phenotype so far (see Discussion).

## Discussion

In this study, 65 unrelated index cases affected with a suspected hereditary podocytopathy, ciliopathy or CAKUT, in which molecular genetic diagnostics had been performed, were examined for a skeletal phenotype. All three disease entities comprise a broad monogenic spectrum with variable clinical phenotypes. In this cohort, eight index cases (12%) were affected by a combination of both renal and skeletal anomalies.

Almost every fifth individual with CAKUT in this study presented with an additional skeletal phenotype. Stoll et al. investigated extrarenal manifestations in children born with CAKUT over a period of 26 years and could observe skeletal anomalies in 97/1678 (6%) individuals (Stoll et al., 2014).

Furthermore, skeletal anomalies like limb defects in individuals with urinary tract malformations have been observed with an incidence of 1:20.000 live births (Natarajan et al., 2013).

Ciliopathies include a wide range of syndromal diseases and can be accompanied by various extrarenal manifestations such as retinal degeneration (Senior-Løken syndrome), vermis hypoplasia, ataxia, and intellectual disability (Joubert syndrome), or occipital meningoencephalocele (Meckel's syndrome) (Hildebrandt et al., 2011). SA are well-known as an extrarenal manifestation, e.g., with Jeune syndrome, Sensenbrenner syndrome, and short-rib polydactyly syndrome (Bredrup et al., 2011). In the present study, the index individual of Family 6 affected by a ciliopathy and SA was genetically diagnosed with a Bardet-Biedl syndrome. Individuals with Bardet-Biedl syndrome show renal anomalies in 53–85% and polydactyly in 77% (Rudling et al., 1996; Forsythe et al., 2018).

The combination of a renal and skeletal phenotype in individuals with an underlying hereditary podocytopathy has mainly been described within Schimke immunoosseous dysplasia (MIM 242900) (Haffner and Petersen, 2019). ES of the index in this study cohort (Family 2; HN-F51) revealed a likely pathogenic variant in *MAFB*. This finding lead to the very rare diagnosis of autosomal dominant MTCO. Zankl et al. published a study comprising 11 index cases with MTCO. Every individual had skeletal anomalies, eight also had an additional renal phenotype consisting of non-specific glomerulosclerosis and severe tubulointerstitial fibrosis (Zankl et al., 2012).

The percentage of individuals with a skeletal and renal phenotype in this study cohort seems rather high (8/65, 12%). However, given the low absolute number of cases (*n* = 8) with a skeletal and renal phenotype, this finding has to be interpreted with caution. In order to achieve a clear statement on the prevalence of SA in hereditary podocytopathies, ciliopathies and (monogenic) CAKUT, the study population for each disease entity would have to be extended. Nevertheless, it is worth mentioning that, at least in this small cohort, almost every fifth individual (5/27, 19%) with CAKUT presented with an additional skeletal anomaly, which is much higher compared to the other subgroups and should encourage investigation of SA as extrarenal manifestation in CAKUT.

It has to be mentioned that the present study has several limitations: (i) the study cohort was small, therefore no assumptions on frequency of renal and skeletal anomalies in CAKUT, hereditary podocytopathies, and ciliopathies can be drawn (see above); (ii) although comprehensive in most cases, the molecular genetic testing strategy is inconsistent as only in 88% of the individuals ES was performed. Nonetheless, all individuals with an additional skeletal phenotype were examined with ES. Therefore, no bias concerning the diagnostic yield should be seen in individuals with skeletal anomalies; and (iii) haploinsufficiency of *SIX2* is not known to be associated with a renal phenotype so far but SIX2 is described to be involved as a key factor within the kidney mesenchyme (Kobayashi et al., 2008; Henn et al., 2018). Therefore, even though the heterozygous deletion of the complete exonic regions of *SIX2* in Family 4 can be classified as pathogenic, its contribution to the renal phenotype is unknown to date.

In conclusion, this study should highlight the association of renal and skeletal phenotypes and their genetic heterogeneity and prompt further systematic and large-scale investigation of the combination of these phenotypic features.

## Data Availability Statement

The datasets presented in this study can be found in online repositories. The names of the repository/repositories and accession number(s) can be found in the article/Supplementary Material.

## Ethics Statement

The studies involving human participants were reviewed and approved by Ethics Committee of the Technical University of Munich, Munich, Germany. Written informed consent to participate in this study was provided by the participants' legal guardian/next of kin. Written informed consent was obtained from the individual(s), and minor(s)' legal guardian/next of kin, for the publication of any potentially identifiable images or data included in this article.

## Author Contributions

MSt, KR, and JH collected all data, performed mutational analysis, and were responsible for writing and revision of the manuscript. BL-S, MG, MBr, RG, MBa, MSc, PS, VT, CS, LR, and UH cared for the patients and provided the clinical data. All authors contributed to the article and approved the submitted version.

## Conflict of Interest

The authors declare that the research was conducted in the absence of any commercial or financial relationships that could be construed as a potential conflict of interest.
